# Swarm-Based Medicine

**DOI:** 10.2196/jmir.2452

**Published:** 2013-09-19

**Authors:** Paul Martin Putora, Jan Oldenburg

**Affiliations:** ^1^Department of Radiation OncologyKantonsspital St GallenSt GallenSwitzerland; ^2^National Resource Center for Late EffectsDepartment of OncologyNorwegian Radium Hospital and Oslo UniversityOsloNorway

**Keywords:** swarm, evidence, eminence, guidelines, recommendations

## Abstract

Occasionally, medical decisions have to be taken in the absence of evidence-based guidelines. Other sources can be drawn upon to fill in the gaps, including experience and intuition. Authorities or experts, with their knowledge and experience, may provide further input—known as “eminence-based medicine”. Due to the Internet and digital media, interactions among physicians now take place at a higher rate than ever before. With the rising number of interconnected individuals and their communication capabilities, the medical community is obtaining the properties of a swarm. The way individual physicians act depends on other physicians; medical societies act based on their members. Swarm behavior might facilitate the generation and distribution of knowledge as an unconscious process. As such, “swarm-based medicine” may add a further source of information to the classical approaches of evidence- and eminence-based medicine. How to integrate swarm-based medicine into practice is left to the individual physician, but even this decision will be influenced by the swarm.

## Introduction

Medicine has become so complex that it is impossible for an individual to fully master even his or her own subspecialty. Physicians need to source information effectively in order to treat their patients according to current state-of-the-art approaches.

With the increasing complexity of medicine, the term “evidence-based medicine” has arisen. This reflects a scientific and arguably objective view of the matter. The highest level of evidence is obtained from randomized trials proving or disproving hypotheses. Generally, evidence-based medicine aims to be universal and is taught as the highest standard in medical schools. When adhering to evidence-based medicine, the responsibility for decisions is transferred from the individual to evidence, which might add to its popularity.

There are many concerns about the limitations of evidence-based medicine, but there are two obvious ones. First, evidence-based medicine is based on available data. However, relevant data is not available for all relevant issues. Also, trials with a negative outcome are underrepresented in medical literature, rendering the evidence base biased and the view of reality skewed [1].

The second obvious flaw is perfectly demonstrated by the parachute study. In this, the authors argue that there is no Level I evidence to support the practice of using parachutes when falling from high altitudes; only common sense and “case reports” suggest this [2]. Thus, the existing data does not pass the stringent standards of evidence-based medicine. In other words, we are and will not be able to make every decision based on available evidence.

The other extreme, to put it bluntly, is “eminence-based medicine”. In this approach, decisions are taken by experts, who unfortunately are sometimes wrong. This form of decision making is predominantly based on clinical experience and a subjective interpretation of the matter. The obvious advantage is that a wider field of decision making can be covered, since the domain is not limited to available evidence. At the same time, errors of individuals may negatively influence decisions. There is no objective control over the process and the drawbacks are obvious.

## Swarm-Based Medicine

The ongoing expansion of information technology is accompanied by a no less remarkable increase in communication supporting medical decision making. With the help of current tools, we are able to communicate faster, on multiple levels, independent of location. With the help of information technology, the medical community is becoming a dynamic, communicating collective with the features of a swarm.

The medical community, with its exponentially growing interactions, is gradually gaining attributes of swarms with inherent intelligence defined as the collective behavior of decentralized, self-organized systems, natural or artificial [3]. A new innovation or change in practice is communicated from members of the swarm such as physicians, hospitals, and medical societies, informally and immediately, rendering the swarm able to follow improved procedures.

Although there is no formalized *typical* procedure of a community, attempts are being made to identify common approaches (eg, with patterns of care studies). They provide valuable feedback on what is happening and help us recognize trends [4].

The National Comprehensive Cancer Network (NCCN) has implemented NCCN Trends, which is a survey-based data and analysis tool. This is being used to gather information from over 200,000 clinicians in order to assess how cancer care is being delivered as well as assess the understanding and acceptance of emerging treatments [5]. This is just one of many projects that are trying to gather insight into knowledge beyond evidence. Most often their focus is not on individuals leading the way, but on the swarm as a whole.

The understanding of the swarm delivering health care is important for several reasons. It means that individuals within the health care system, though basically the same, do not perform identical actions and the effect of health care on the population is determined by the swarm’s behavior rather than by solitary outliers.

Trials may be coordinated among multiple nations; information from conferences as well as its critical appraisal by experts are available online. Medical societies as well as individuals interact with social media. Physicians usually incorporate input from evidence and eminence. Medical literature as well as practical tips and tricks from colleagues during a coffee break are both essential. As innovative IT methods are increasingly utilized in registries (eg, cancer registries) and faster tools for patterns of care studies are being implemented, the old “coffee break tips” are enhanced by information from the community—from the swarm.

Although not a conscious process, the pure mass of the “normal” serves as quality assurance, as a reference of what is typical as well as a form of benchmarking against which to compare one’s own views. In comparison to randomized trials, the community practice patterns—the decisions of the swarm—undergo everyday scrutiny and have to perform out in the *wild*.

In the classical swarm model as introduced by Reynolds, the swarm consists of “boids” [6], which are agents that follow simple rules and are not aware of the product of swarm intelligence. Swarm-based models have been implemented on a lower level in medicine (eg, the Ant Colony Optimization algorithm in radiotherapy planning); however, their application on a social level is more complex [7]. By conscious interaction, agents executing treatment decisions face information on the swarm’s behavior. It has been demonstrated that this knowledge does not obstruct crowd wisdom [8].

One example of the balancing of crowd intelligence and eminence-based medicine might be the process of electronic voting (eVote), during the European Consensus Conference on Diagnosis and Treatment of Germ-Cell Cancer, on areas without sufficient evidence for decision-making [9]. The “swarm” of 60 germ cell cancer experts, mostly from Europe but also from the United States and Canada, had the opportunity to eVote on 48 areas of considerable controversy, caused by lack of evidence.

Intriguingly, the most unanimous eVotes were achieved after distinguished experts had argued in favor of a particular approach. However, participants did not follow recommendations that, during lively discussions, were perceived as unconvincing or unreasonable. “Vox Populi”, or crowd wisdom, and swarm behavior probably follow similar principles and might be greatly facilitated by modern digital communication.

The availability of information is essential in the creation of guidelines. An interesting comparison of guidelines from 13 countries on lower back pain revealed many similarities [10]. The authors observed that the differences were few and probably less than might be expected for different healthcare systems and cultures, likely due in part to guideline committees usually being aware of the content of other guidelines and being motivated to produce similar recommendations. In some instances, the guidelines were national adaptations of European guidelines. Similarly, several investigations, such as the European Initiative on Quality Management in Lung Cancer Care [11], have found many similarities among guidelines on lung cancer care, although the quality of them varied significantly. As these guidelines were based on available literature and previously presented guidelines, this led to a large overlap of cited sources.

## Impact of Swarm-Based Medicine

Humans, armed with Internet technology, exercise crowd intelligence in various spheres of social interaction ranging from predicting elections to company management [12]. Internet-based interaction may result in different outcomes, such as improved response capability and decision-making quality.

The direct comparison of swarm-based medicine with evidence- or eminence-based is interesting, but these concepts should be perceived as complementing each other and working independently of each other. Optimal decision making depends on a balance of personal knowledge and swarm intelligence, taking into account the quality of each, with their weight in decisions being adapted accordingly [13]. The possibility of balancing controversial standpoints and achieving acceptable conclusions for the majority of participants has been an important task of scientific and medical conferences since the Age of Enlightenment in the 17th and 18th centuries. Our swarm continues with this interconnecting synchronization at an unprecedented speed and is, thanks to eVotes, Internet forums, and the like, more reactive than ever. Faster changes in our direction of movement, like a school of fish, are becoming possible. Information spreads from one individual to another. It is unconscious, but with our own *dance* we influence the rest of the *beehive*.

Within an environment, individual behavior determines the behavior of the collective and vice versa. Internet technology has dramatically changed the environment we behave in. Traditionally, medical information was provided to patients as well as to physicians by experts. This intermediation was characterized by an expert standing between sources of information and the user. Currently, and probably even more so in the future, Web 2.0 and appropriate algorithms enable users to rely on the guidance or behavior of their peers in selecting and consuming information. This is one of many processes facilitated by medicine 2.0 and is described as “apomediation” [14]. Apomediation, whether implicit or explicit, increases the influence of individuals on others. For an individual to adapt its behavior within a swarm, other individuals need to be perceived and their actions reacted upon. Through apomediation, more individuals take part in the swarm.

Our patients are better informed; second opinions can be sought via the Internet within hours. Our individual behavior is influenced by online resources as well as digital communication with our colleagues. This change in individual behavior influences the way we find, understand, and adopt guidelines. Societies representing larger groups within the swarms use this technology to create recommendations. This process is influenced by individuals and previous actions of the community; these then in return influence individual behavior. Information technology has a major impact on the lifecycle of guidelines and recommendations. There is no entry and exit point for IT in this regard. With increasing influence on individual behavior, its influence on collective behavior increases, influencing the other direction to the same extent.

Dynamic changes in movement of the swarm and within the swarm may lead to individuals leaving the herd. These may influence the herd to move in the direction of the outliers. At the same time, an individual leaving a flock or swarm is exposed. Physicians as well as clinical centers expose themselves when they leave the group for the sake of innovation. Negative results and failure might lead to legal exposure should treatments fail.

The perception of swarm behavior itself changes the way we approach guidelines. When several guidelines are published, being aware of them as a result of interaction increases our awareness for bias. Major deviations from other recommendations warrant scrutiny. The perception of swarm behavior and embracing the knowledge of the swarm may lead to an optimized use of resources. Information that has already been obtained may be incorporated directly by agents, enabling them to build on this and establish new knowledge—as social learning agents [15,16].

## Conclusion

Swarm behavior might facilitate the generation and distribution of knowledge. Swarm behavior may also be detrimental. Some innovations may be hindered as they might be perceived as outliers. However, this negative effect may also be partially counteracted by a conscious perception of the swarm aspect of our behavior.

The amount of collected data is increasing exponentially and data mining and recommender systems are improving in parallel. These new tools will provide us with information on our collective behavior, which was not accessible until now. As with many valuable sources, it will be interesting to see how they will be approached by academia and industry.

This information does not need be produced; it lies before us and all we need to do is become aware of it. How to integrate swarm-based medicine into practice is left to the individual physician, but even this decision will be influenced by the swarm.
